# Diversity and Inclusion in Internal Medicine Training Programs: An Unfulfilled Dream

**DOI:** 10.7759/cureus.21974

**Published:** 2022-02-07

**Authors:** Hamza Maqsood, Shifa Younus, Sadiq Naveed, Aftab Ahmad, Ateeq U Rehman, Faisal Khosa

**Affiliations:** 1 Internal Medicine, Nishtar Medical University, Multan, PAK; 2 Psychiatry, Hartford Hospital, Institute of Living, Hartford, USA; 3 Internal Medicine, Orange Park Medical Center, Orange Park, USA; 4 Internal Medicine, Mercer University School of Medicine, Macon, USA; 5 Internal Medicine, Marshfield Clinic Health System, Marshfield, USA; 6 Radiology, Vancouver General Hospital, Vancouver, CAN

**Keywords:** medical residency, racial disparity, internal medicine, healthcare, gender disparity

## Abstract

Background

Promoting a diversified healthcare force fosters more culturally centered care, expands the approach to high-quality healthcare for poorly served populations, improves patient contentment, and broadens research agendas, all components essential to minimize healthcare imbalances. Our study reviews the trends of gender and racial disparity in Internal Medicine residency programs.

Methodology

In this retrospective analysis, we extracted data from the Accreditation Council for Graduate Medical Education’s annual Data Resource Books from 2007 to 2019. Gender was reported as males and females. Race/ethnicity was cataloged as White/non-Hispanic, Black/non-Hispanic, Hispanic, Asian or Pacific Islander, Native American/Alaskan, others, and unknown.

Results

The representation of women increased progressively, with a relative increase of 4.7% from 2007 to 2019. For race/ethnicity, the study period started from the year 2011. When averaged across the eight-year study period, 27% of the study sample were White (non-Hispanic), followed by Asian/Pacific Islanders at 21%. The representation of other races was even lower. For 36.2% of the residents, the racial data were not known and categorized as unknown racial distribution.

Conclusions

Our study reports that gender and racial/ethnic imbalance persists within the training programs of Internal Medicine. Effectual strategies should be implemented to improve access to care to the underrepresented communities, address physician shortages in different areas of the country, and strengthen our ability to address long-established disparities in healthcare and outcomes.

## Introduction

Fostering a diverse healthcare workforce promotes more culturally responsive care, improves access to high-quality healthcare for underserved populations, increases patient satisfaction, and broadens research agendas, all components necessary to eliminate healthcare disparities [1]. Today, modern medicine is facing several challenges. Implicit and explicit bias, systemic racism, and sexism affect our communities [2]. Overall, there has been gradual interest in promoting and teaching diversity. Institutions have been devising policies and administrative positions fostering inclusion and diversity over the last decade [2]. However, diversity training has so far failed to objectively increase the representation and advancement of females and minority groups in healthcare [1,2].

A progressive approach toward more balanced gender and racial distributions has been observed during medical school admissions; however, disparities remain in academic medicine [3,4]. Racial minority groups, such as Hispanic and Black/African American physicians, have lesser opportunities than the White faculty, especially in lead roles [4,5]. Likewise, female physicians are disadvantaged in faculty promotions, leading to only 13% of department heads at well-known US medical schools [6]. These visual representations of the medical culture reinforce the acceptable norms and values, White and masculine, in medicine. This disproportionate representation is discernible across multiple subspecialties of medicine, National Institutes of Health funding, professional societies, medical journals’ editorial boards, and clinical trials [7-12].

Similar gender and racial disparity persist in the specialty of Internal Medicine [13]. Black/African American and female general internists have reported lower annual incomes [14]. In addition, a study analyzed the profiles of medical school enrollees, graduates, and Internal Medicine faculty and showed notable disparities for Black/African Americans and multiple race physicians [15]. They also reported that more than half of all physicians were men (63.6%). This rift was more striking for higher ranks which were majorly occupied by males [15]. These findings are comparable to another study probing the trends in Internal Medicine faculty by sex and race/ethnicity [16].

There is an ethical imperative to work toward the representation of more diversified backgrounds in the Internal Medicine workforce. Promoting diversity will maximize our ability to include and communicate with patients of all backgrounds, particularly minorities, and may improve the quality of healthcare [15,17]. Our study aims to characterize the gender and racial trends among Internal Medicine residents.

## Materials and methods

In this retrospective analysis, we extracted the data from the annual Accreditation Council for Graduate Medical Education’s Data Resource Books from 2007 to 2019 [18].

Variables

For Internal Medicine, demographic data (i.e., race/ethnicity and gender) of residents were extracted. Gender was reported as males, females, and not reported. Race/ethnicity was cataloged as White/non-Hispanic, Black/non-Hispanic, Hispanic, Asian or Pacific Islander, Native American/Alaskan, others, and unknown.

Data analysis

The data were analyzed based on the gender and racial distributions and their temporal trends by year among Internal Medicine residents. Relative and absolute percentages were calculated along with counts to underline trends in resident appointments over time and across these specialties. Although data for gender distribution were available for all years (i.e., 2007 to 2019), race/ethnicity was reported from 2011.

## Results

**Figure 1:**
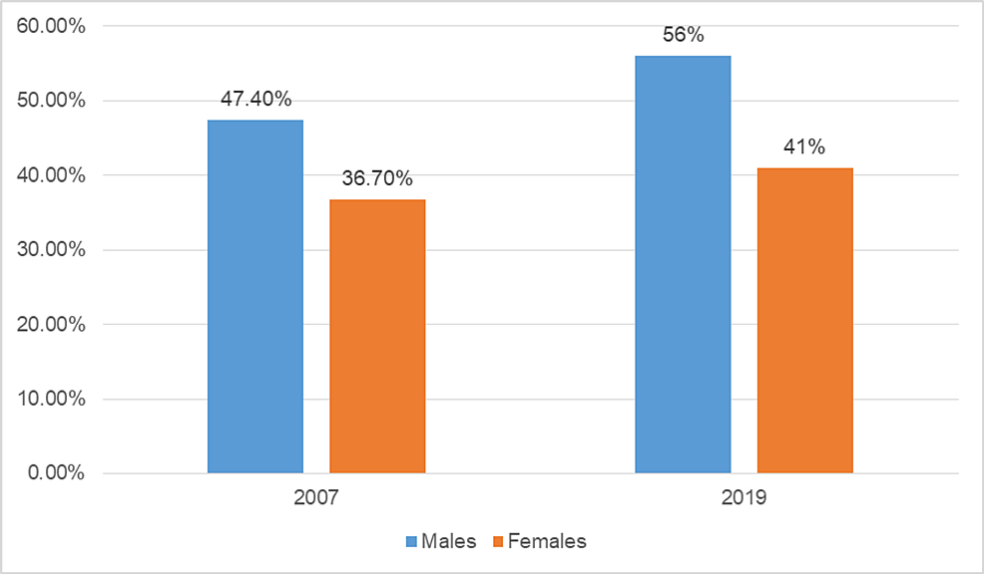
Gender differences at the beginning and end of our study period (i.e., 2007 to 2019).

When averaged over our study period, 51.4% of all Internal Medicine residents were men, whereas the representation of women was 38.3% (p < 0.001). Gender data were not reported by 10% of the residents. The representation of women increased steadily, with a relative increase of 4.7% from 2007 to 2019. However, the rate of growth for our study period was much greater for men than women. In 2007, men accounted for 47.4% while women accounted for 36.7% of all residents in Internal Medicine, whereas in 2019, men accounted for 56% while women accounted for 41% of all academic Internal Medicine residents (Figure 1).

For analysis of racial distribution, our study period ranged from 2011 to 2019. When averaged across the eight-year study period, 27% of the study sample was White (non-Hispanic), followed by Asian/Pacific Islanders at 21%. The representation of Hispanics was 5%, Black/African Americans was 4.2%, Native Americans/Alaskans was 0.14%, and others was 6.4% of the total study population. For 36.2% of the residents, racial data were not known and categorized as unknown racial distribution.

The absolute change in racial distribution was the highest for Whites (+10), followed by Asian/Pacific Islanders (+4.3), Hispanics (+1.7), Black/African Americans (+1), Native Americans/Alaskans (+0.08), and others (+1.6) (Table 1).

**Table 1:** Gender and racial differences as well as absolute and relative changes at the start and end of our study period.

	2011 (%)	2019 (%)	Absolute change (%)
Race/Ethnicity			
White	23	32.8	+9.8
Asian/Pacific Islander	21	25.3	+4.3
Hispanic	4.6	6.3	+1.7
Black/African Americans	4	5	+1
Native Americans/Alaskans	0.12	0.2	+0.08
Others	6.8	8.4	+1.6
Unknown	40	22	−18
Gender			
Males	47.4	56	+8.6
Females	36.7	41	+4.3

The yearly percentage of all Internal Medicine residents by race and gender is shown in Table 2.

**Table 2:** Temporal trends for race and gender and absolute percentage change from the year 2007 to 2019.

	2007 (%)	2008 (%)	2009 (%)	2010 (%)	2011 (%)	2012 (%)	2013 (%)	2014 (%)	2015 (%)	2016 (%)	2017 (%)	2018 (%)	2019 (%)
White					23	24.3	24.8	25	25.8	27.1	28.3	28	32.8
Asian/Pacific Islander					21	21.3	21.1	20.7	20.6	21	21	21.3	25.3
Hispanic					4.6	4.3	4.4	4.5	4.8	05	05	5.3	6.3
Black					04	4.2	4.2	4.1	04	3.8	4.1	4.2	05
Native American/Alaskan					0.12	0.12	0.18	0.2	0.16	0.1	0.08	0.1	0.2
Others					6.8	6.6	6.2	5.8	5.6	5.8	6.1	6.5	8.4
Unknown					40	38.8	40	39.7	39	37	35.3	34.4	22
Male	47.4	47	46.4	47	46.2	50	51.5	53.6	53.6	53.8	54.2	54.5	56
Female	36.7	36.8	37	37.7	37.1	38.5	39.5	40.4	40.6	40	40	40	41
Not reported	15.8	16.2	16.1	15	16.5	11.4	09	06	5.7	6.3	06	5.7	02

## Discussion

We explored the distribution of gender among residents in Internal Medicine over 12 years from 2007 to 2019 and the racial/ethnic distribution over eight years from 2011 to 2019. When averaged across the 12 years of the study period, almost 51% of all Internal Medicine residents were men while the representation of women was only 38%; these findings are consistent with those of previous studies [15,16]. In our study, female residents increased in proportion, and their representation increased from 36.7% in 2007 to 41% in 2019. These findings are consistent with existing literature, suggesting a dissatisfactory increase in the percentage of female residents in Internal Medicine [15,16].

Both male and female faculty share similar notions of being involved and dedicated about their work and have a similar inclination toward leadership positions. Yet, the confidence level about career advancement in females is not comparable to male physicians. Female physicians do not experience equivalent inclusion in the environment of academia [19]. Discrepancies in mentoring, opportunities, or conscious or unconscious bias on the part of residents and faculty are the basis of the underrepresentation of women in internal medicine [20]. Ely and Meyerson examined how organizational culture centers primarily on men’s needs and expectations. This study also suggested that the marginalization can also be a product of indigenous gender bias in both the implicit and explicit practices of the organization [21]. Our findings are conclusive of similar patterns of gender inequity in internal medicine. Burgess et al. performed a study and revealed archetypal conditions for female physicians to be subject to stereotype threat, promoting declining self-confidence and potential [22]. These elements, along with curtailed self-efficacy, isolation, unconscious bias, lack of sponsorship, lack of early discipline-related exposure, and work-life balance, likely contribute to underrepresentation and the slow pace of professional progression for female physicians in Internal Medicine [20].

A comprehensive plan to advance and reinforce women’s careers in Internal Medicine should be formulated at all levels. The cultural shift should hold departments liable for drafting and encouraging women, employing diversity officials, voluntary training, cross-training to maximize contact among different groups, and mentoring initiatives that match senior leadership to junior female faculty [23]. In addition, the administration should consider strategy and plan changes, including catering flexible working conditions for female faculty and assistance to help them navigate the hurdles and challenges they face at work [23]. At the national level, incentives should be given for research to recognize and address the roots and sustainers of gender discrimination. Appreciating and publicizing the career achievements of females in Internal Medicine could encourage and inspire women in their early careers to prepare for leadership positions in the future [23].

A parallel bias is seen while comparing the trends of different races/ethnicities within the residency programs of Internal Medicine in the United States. For example, the White/Caucasian race was overrepresented in many years, followed by Asians among all Internal Medicine residents. According to the conclusions of a previous study, the increasing Asian faculty is due to a parallel increase in the total population of Asians in the United States [24]. Other races have even smaller representation in healthcare.

The underrepresentation of minority groups among physicians is a challenging problem with many contributing factors. Lett et al. reported that underrepresented minorities (URMs) are less represented among the candidates applying and the matriculate pool. It is well established that interlinked communal factors such as below-the-bar investment in public education, discrimination in educational material budgeting, and de facto school isolation may hinder the educational opportunities of minority populations, thus potentially brushing aside from the pipeline of these populations to medical institutes [25]. In addition, several other barriers are faced by URMs in getting a training spot and promotions in faculty positions, including a huge debt load, restricted communication skills, racial/ethnic influence, and disparities in the workplace [15,16]. The lack of minority preceptors in Internal Medicine also affects the recruitment of minorities in the field [4]. From a workforce strategy viewpoint, maximizing the representation from Black, Hispanic, and Native American populations, in particular, may help bring down the tenacious geographic misallocation of the overall physician workforce [26,27].

The society of Hospital Medicine is already devising strategies to encourage diversity, equity, and inclusion. These include issuing a formal Diversity and Inclusion Statement, creating the Diversity, Equity, and Inclusion Special Interest Group (DEI SIG), and recently forming a board-designated DEI task force charged with making suggestions to maximize diversity in Hospital Medicine. In addition, existing literature has highlighted specific recommendations to minimize the racial disparity in healthcare in general and Internal Medicine specifically. Various mentoring programs along with formal and structured advocacy should be created [28]. Public relations campaigns should be undertaken to highlight to health systems and other employers the underrepresentation of Black and Latino hospitalists [28]. Finally, we should focus on “building the pipeline,” which means increasing the number of people from one’s community who are nurtured academically and socially not only to pursue careers in science and medicine but also to be determined candidates and accomplished students, trainees, and physicians [29,30]. The findings of this study conclude that further research is needed into the composite etiologies leading to the declining representation of URM in residency programs of Internal Medicine.

Limitations

Our study has its share of limitations. This study focused on data on residents of Internal Medicine and thus may have limited generalizability to other subspecialties. Throughout our study period, a significant chunk of residents had non-specified gender and racial identity, ultimately leading to variations in findings. Our study is based on a dataset that describes gender in a binary fashion. Finally, our study did not explore the combined effects of being a gender and a racial minority, such as female Hispanic or Black female residents.

## Conclusions

Our study concludes that gender and racial disparity persist within residency programs in Internal Medicine. A comprehensive approach involving multilevel efforts is required to provide greater support for females and for the careers of URM faculty to ensure their unbiased representation at all levels of academic medicine. Effective policies at all three key stakeholders levels should be implemented, which include educational institutions, national associations representing those educational institutions, and state policymakers. A diverse workforce will improve access to care to the URM communities, help address physician shortages in different areas of the country, and ameliorate our propensity to address longstanding disparities in healthcare.
